# Supplementary material for: Weekend effect in upper gastrointestinal bleeding: a systematic review and meta-analysis
Source: PeerJ. 2018 Jan 12;6:e4248. doi: 10.7717/peerj.4248 (PMC5768163; doi:10.7717/peerj.4248)
Supplement: Table S2 [file peerj-06-4248-s002.docx]

**Search strategy**

PUBMED

| Search | Query | Items found |
| --- | --- | --- |
| #17 | Search ((((((Peptic Ulcer Hemorrhage) OR Peptic Ulcer bleeding) OR upper Gastrointestinal Hemorrhage) OR upper Gastrointestinal bleeding) OR (((((Upper Gastrointestinal Tract OR Upper GI Tract OR Upper Gastrointestinal OR Duodenum OR Esophagus OR esophag* OR Stomach)) OR ((Esophageal and Gastric Varices OR Esophageal Varices OR Esophageal Varix OR Gastric Varix OR gastrointestinal variceal)))) AND ((Hemorrhage OR bleeding OR bleed*))))) AND ((holidays) OR ((weekend* OR nighttime OR admission time OR off hours OR off-hours OR admitted time))) | 378 |
| #16 | Search (((((((Peptic Ulcer Hemorrhage) OR Peptic Ulcer bleeding) OR upper Gastrointestinal Hemorrhage) OR upper Gastrointestinal bleeding) OR (Upper Gastrointestinal Tract OR Upper GI Tract OR Upper Gastrointestinal OR Duodenum OR Esophagus OR esophag* OR Stomach)) OR ((((((Esophageal and Gastric Varices OR Esophageal Varices OR Esophageal Varix OR Gastric Varix OR gastrointestinal varicea*)) OR (esophageal and gastric varices OR esophageal varices OR esophageal varix OR gastric varix OR gastrointestinal varices)) OR variceal)) AND ((Hemorrhage OR bleeding OR bleed*))))) AND ((holidays) OR ((weekend* OR nighttime OR admission time OR off hours OR off-hours OR admitted time))) | 2173 |
| #15 | Search (holidays) OR ((weekend* OR nighttime OR admission time OR off hours OR off-hours OR admitted time)) | 90882 |
| #14 | Search (weekend* OR nighttime OR admission time OR off hours OR off-hours OR admitted time) | 87158 |
| #13 | Search holidays | 4263 |
| #12 | Search (((((Peptic Ulcer Hemorrhage) OR Peptic Ulcer bleeding) OR upper Gastrointestinal Hemorrhage) OR upper Gastrointestinal bleeding) OR (Upper Gastrointestinal Tract OR Upper GI Tract OR Upper Gastrointestinal OR Duodenum OR Esophagus OR esophag* OR Stomach)) OR ((((((Esophageal and Gastric Varices OR Esophageal Varices OR Esophageal Varix OR Gastric Varix OR gastrointestinal varicea*)) OR (esophageal and gastric varices OR esophageal varices OR esophageal varix OR gastric varix OR gastrointestinal varices)) OR variceal)) AND ((Hemorrhage OR bleeding OR bleed*))) | 486358 |
| #11 | Search (((((Esophageal and Gastric Varices OR Esophageal Varices OR Esophageal Varix OR Gastric Varix OR gastrointestinal varicea*)) OR (esophageal and gastric varices OR esophageal varices OR esophageal varix OR gastric varix OR gastrointestinal varices)) OR variceal)) AND ((Hemorrhage OR bleeding OR bleed*)) | 59347 |
| #10 | Search (Hemorrhage OR bleeding OR bleed*) | 458261 |
| #9 | Search (((Esophageal and Gastric Varices OR Esophageal Varices OR Esophageal Varix OR Gastric Varix OR gastrointestinal varicea*)) OR (esophageal and gastric varices OR esophageal varices OR esophageal varix OR gastric varix OR gastrointestinal varices)) OR variceal | 334296 |
| #8 | Search variceal | 6536 |
| #7 | Search esophageal and gastric varices OR esophageal varices OR esophageal varix OR gastric varix OR gastrointestinal varices | 15485 |
| #6 | Search Esophageal and Gastric Varices OR Esophageal Varices OR Esophageal Varix OR Gastric Varix OR gastrointestinal varicea* | 332826 |
| #5 | Search Upper Gastrointestinal Tract OR Upper GI Tract OR Upper Gastrointestinal OR Duodenum OR Esophagus OR esophag* OR Stomach | 451323 |
| #4 | Search upper Gastrointestinal bleeding | 18357 |
| #3 | Search upper Gastrointestinal Hemorrhage | 15025 |
| #2 | Search Peptic Ulcer bleeding | 10217 |
| #1 | Search Peptic Ulcer Hemorrhage | 9204 |

COCHRANE

| Search | Query | Items found |
| --- | --- | --- |
| #1 | MeSH descriptor: [Peptic Ulcer Hemorrhage] explode all trees | 450 |
| #2 | Peptic Ulcer Hemorrhage or Peptic Ulcer bleeding or upper Gastrointestinal Hemorrhage or upper Gastrointestinal bleed*:ti,ab,kw (Word variations have been searched) | 1711 |
| #3 | ugib or ugi bleeding:ti,ab,kw (Word variations have been searched) | 56 |
| #4 | #1 or #2 or #3 | 1722 |
| #5 | MeSH descriptor: [Upper Gastrointestinal Tract] explode all trees | 4337 |
| #6 | Upper Gastrointestinal Tract or Upper GI Tract or Upper Gastrointestinal or Duodenum or Esophagus or esophag* or Stomach or Esophageal and Gastric Varices or Esophageal Varices or Esophageal Varix or Gastric Varix or gastrointestinal variceal:ti,ab,kw (Word variations have been searched) | 23403 |
| #7 | #5 or #6 | 24275 |
| #8 | MeSH descriptor: [Hemorrhage] explode all trees | 12049 |
| #9 | Hemorrhage or bleeding or bleed*:ti,ab,kw (Word variations have been searched) | 35459 |
| #10 | #8 or #9 | 37771 |
| #11 | #7 and #10 | 3406 |
| #12 | #4 or #11 | 3913 |
| #13 | holidays or holiday* or nighttime or night time or admission time or admitted time or off hours or off-hours:ti,ab,kw (Word variations have been searched) | 15686 |
| #14 | #12 and #13 | 96 |

EMBASE

| Search | Query | Items found |
| --- | --- | --- |
| 1 | exp esophagus hemorrhage/ or esophagus hemorrhage.mp. | 7091 |
| 2 | esophagus ulcer hemorrhage.mp. or exp esophagus ulcer hemorrhage/ | 64 |
| 3 | esophagus varices bleeding.mp. or exp esophagus varices bleeding/ | 4424 |
| 4 | peptic ulcer bleeding.mp. or exp peptic ulcer bleeding/ | 5270 |
| 5 | stomach hemorrhage.mp. or exp stomach hemorrhage/ | 5147 |
| 6 | Duodenum bleeding.mp. or exp duodenum bleeding/ | 1581 |
| 7 | upper Gastrointestinal Hemorrhage.mp. or exp upper gastrointestinal bleeding/ | 7990 |
| 8 | (ugib or ugih or upper gi bleed*).mp. [mp=title, abstract, heading word, drug trade name, original title, device manufacturer, drug manufacturer, device trade name, keyword, floating subheading] | 2530 |
| 9 | exp abdominal bleeding/ | 3642 |
| 10 | gastric ulcer bleeding/ or peptic ulcer bleeding/ or duodenum bleeding/ or upper gastrointestinal bleeding/ or intestinal bleeding/ or duodenal ulcer bleeding/ or stomach varices bleeding/ or esophagus varices bleeding/ | 19085 |
| 11 | Upper Gastrointestinal Tract.mp. or exp upper gastrointestinal tract/ | 9450 |
| 12 | (Upper GI Tract or Upper Gastr* or Duodenum or Esophagus or esophag* or Stomach or Esophageal or Gastric Varices or Esophageal Varices or Esophageal Varix or Gastric Varix or gastrointestinal variceal or varix ulcer).mp. [mp=title, abstract, heading word, drug trade name, original title, device manufacturer, drug manufacturer, device trade name, keyword, floating subheading] | 703683 |
| 13 | (ugi or upper gi).mp. [mp=title, abstract, heading word, drug trade name, original title, device manufacturer, drug manufacturer, device trade name, keyword, floating subheading] | 10520 |
| 14 | 11 or 12 or 13 | 706603 |
| 15 | bleeding.mp. or bleeding/ | 452081 |
| 16 | hemorrhage.mp. | 343640 |
| 17 | bleed*.mp. | 460938 |
| 18 | 15 or 16 or 17 | 662739 |
| 19 | 14 and 18 | 69849 |
| 20 | 1 or 2 or 3 or 4 or 5 or 6 or 7 or 8 or 9 or 10 or 19 | 77243 |
| 21 | holiday.mp. | 3650 |
| 22 | exp night/ or nighttime.mp. | 47356 |
| 23 | night time.mp. | 7386 |
| 24 | admission time.mp. | 939 |
| 25 | admitted time.mp. | 3 |
| 26 | admit* time.mp. | 41 |
| 27 | holiday*.mp. | 6386 |
| 28 | (off hours or off-hours).mp. | 463 |
| 29 | off hour*.mp. | 529 |
| 30 | off-hours.mp. | 463 |
| 31 | 21 or 22 or 23 or 24 or 25 or 26 or 27 or 28 or 29 or 30 | 58075 |
| 32 | 20 and 31 | 164 |

CINAHL

| Search | Query | Items found |
| --- | --- | --- |
| S1 | Peptic Ulcer Hemorrhage | 380 |
| S2 | Peptic Ulcer bleeding | 360 |
| S3 | upper Gastrointestinal bleeding | 616 |
| S4 | "upper Gastrointestinal Hemorrhage" | 37 |
| S5 | "Peptic Ulcer bleeding" | 65 |
| S6 | S1 OR S2 OR S3 OR S4 OR S5 | 1029 |
| S7 | Upper Gastrointestinal Tract OR Upper GI Tract OR Upper Gastrointestinal OR Duodenum OR Esophagus OR esophag* OR Stomach OR Esophageal OR Gastric Varices OR Esophageal Varices OR Esophageal Varix OR Gastric Varix OR gastrointestinal variceal | 17972 |
| S8 | ( (MH "Hemorrhage") OR "bleeding" ) OR bleed* | 19351 |
| S9 | S7 AND S8 | 1330 |
| S10 | S6 OR S9 | 1706 |
| S11 | (MH "Holidays") OR "holiday" | 3610 |
| S12 | weekend* OR nighttime OR admission time OR off hours OR off-hours OR admitted time | 17206 |
| S13 | S11 OR S12 | 20761 |
| S14 | S10 AND S13 | 31 |

Software for screening and/or citation management: EndNote X8.1
