# Supplementary material for: Weekend effect in upper gastrointestinal bleeding: a systematic review and meta-analysis
Source: PeerJ. 2018 Jan 12;6:e4248. doi: 10.7717/peerj.4248 (PMC5768163; doi:10.7717/peerj.4248)
Supplement: Table S3 [file peerj-06-4248-s003.docx]

**NOS assessment**

|  | **Selection** | | | | **Comparability** | | **Outcome** | | | **NOS score** |
| --- | --- | --- | --- | --- | --- | --- | --- | --- | --- | --- |
| **First author, year** | Representativeness of the exposed cohort | Non exposed  drawn from the  same  community  as exposed | Ascertain  exposure  through secure  records or  structured  interviews | Demonstration that outcome of interest was not present at start of study | Study controls for age, sex | Study controls for any additional factors | Assessment of outcome through secure records | Follow up long enough for outcomes to occur | Loss follow up <20% |  |
| **Weeda, 2016** | V | V | V | V | V | V | V | V | V | 9 |
| **Ahmed, 2015** | V | V | V | V |  |  | V | V | V | 7 |
| **Al-Qahatani, 2015** | V | V | V | V | V | V | V | V | V | 9 |
| **Wu, 2014** | V | V | V | V |  |  | V | V | V | 7 |
| **Tufegdzic, 2014** | V | V | V | V |  | V | V | V | V | 8 |
| **Abougergi, 2014** | V | V | V | V | V | V | V | V | V | 9 |
| **Youn, 2012** | V | V | V | V |  | V | V | V | V | 8 |
| **Byun, 2012** | V | V | V | V |  | V | V | V | V | 8 |
| **Tsoi, 2012** | V | V | V | V |  | V | V | V | V | 8 |
| **Haas, 2012** | V | V | V | V |  |  | V | V | V | 7 |
| **Groot, 2012** | V | V | V | V |  | V | V | V | V | 8 |
| **Button, 2011** | V | V | V | V | V | V | V | V | V | 9 |
| **Jairath, 2011** | V | V | V | V | V | V | V | V | V | 9 |
| **Dorn, 2010** | V | V | V | V | V | V | V | V | V | 9 |
| **Ananthakrishnan, 2009** | V | V | V | V | V | V | V | V | V | 9 |
| **Myers, 2009** | V | V | V | V | V | V | V | V | V | 9 |
| **Shaheen, 2009** | V | V | V | V | V | V | V | V | V | 9 |
| **Schmulewitz, 2005** | V | V | V | V | V |  | V | V | V | 8 |
