# Supplementary material for: Weekend effect in upper gastrointestinal bleeding: a systematic review and meta-analysis
Source: PeerJ. 2018 Jan 12;6:e4248. doi: 10.7717/peerj.4248 (PMC5768163; doi:10.7717/peerj.4248)
Supplement: Table S4 [file peerj-06-4248-s004.docx]

| First author, year | Weekday  admissions | Weekend  admissions | Total No. of patients |
| --- | --- | --- | --- |
| **Weeda, 2016** | **UGIB** | | |
| No. of patients | 90102 | 29251 | 119353 |
| No. of patients died in hospital | 1990 | 707 |  |
|  | **Variceal bleeding** | | |
| No. of patients | 8156 | 2766 |  |
| No. of patients died in hospital | 414 | 147 |  |
|  | **Non-variceal bleeding** | | |
| No. of patients | 81946 | 26485 |  |
| No. of patients died in hospital | 1576 | 560 |  |
| **Al-Qahatani, 2015** | **Variceal bleeding** | | |
| No. of patients | 685 | 252 | 937 |
| No. of patients died in hospital | 47 | 13 |  |
| Time to endoscopy Mean/SD (hours) | 7.56/7.8 | 9/2.32 |  |
| **Wu, 2014** | **Non-variceal bleeding** | | |
| No. of patients | 615 | 129 | 744 |
| No. of patients died in hospital | 84 | 15 |  |
| Time to endoscopy Mean/SD (hours) | 16.7/19.8 | 12.2/15.3 |  |
| **Tufegdzic, 2014** | **UGIB** | | |
| No. of patients | 318 | 175 | 493 |
| No. of patients died in hospital | 19 | 12 |  |
|  | **Variceal bleeding** | | |
| No. of patients | 18 | 16 | 34 |
| No. of patients died in hospital | 2 | 4 |  |
|  | **Non-variceal bleeding** | | |
| No. of patients | 300 | 159 | 459 |
| No. of patients died in hospital | 17 | 8 |  |
| **Abougergi, 2014** | **UGIB** | | |
| No. of patients | 153052 | 49207 | 202259 |
| No. of patients died in hospital | 3230 | 1141 |  |
|  | **Variceal bleeding** | | |
| No. of patients | 2463 | 788 | 3251 |
| No. of patients died in hospital | 147 | 47 |  |
|  | **Non-variceal bleeding** | | |
| No. of patients | 150589 | 48419 | 199008 |
| No. of patients died in hospital | 3083 | 1093 |  |
| **Youn, 2012** | **Non-variceal bleeding** | | |
| No. of patients | 326 | 62 | 388 |
| No. of patients died in hospital | 6 | 1 |  |
| Time to endoscopy Mean/SD (hours) | 5.6/0.4 | 5.1/0.8 |  |
| **Byun, 2012** | **Variceal bleeding** | | |
| No. of patients | 220 | 74 | 294 |
| No. of patients died in hospital | 48 | 17 |  |
| Time to endoscopy Median(range) (hours) | 7.5(0.1-305.3) | 11.3(1.0-355.7) |  |
| **Tsoi, 2012** | **Non-variceal bleeding** | | |
| No. of patients | 6649 | 1573 | 8222 |
| No. of patients died within 30 days | 269 | 65 |  |
| Time to endoscopy Mean/SD (hours) | 23.3/38.4 | 23.5/43.2 |  |
| **Haas, 2012** | **UGIB** | | |
| No. of patients | 124 | 50 | 174 |
| No. of patients died in hospital | 1 | 0 |  |
| No. of patients died within 30 days | 4 | 0 |  |
| Time to endoscopy Mean/SD (hours) | 10.8/9.3 | 7.5/7.0 |  |
| **Groot, 2012** | **UGIB** | | |
| No. of patients | 404 | 167 | 571 |
| No. of patients died within 30 days | 12 | 15 | 27 |
| **Button, 2011** | **UGIB** | | |
| No. of patients | 18285 | 6136 | 24421 |
| No. of patients died within 30 days | 1792 | 660 | 2452 |
| **Jairath, 2011** | **UGIB** | | |
| No. of patients | 5250 | 1499 | 6749 |
| No. of patients died in hospital | 527 | 148 |  |
| Time to endoscopy Median(25th-75th percentile) (hours) | 21.8(11.3-47.7) | 38.9(15.8- 64.2) |  |
| **Dorn, 2010** | **UGIB** | | |
| No. of patients | 75636 | 23339 | 98975 |
| No. of patients died in hospital | 2496 | 887 |  |
| Time to endoscopy Mean/SD (hours) | 67.2/36 | 74.4/33.6 |  |
| **Ananthakrishnan, 2009** | **UGIB** | | |
| No. of patients | 322937 | 97002 | 419939 |
| No. of patients died in hospital | 11503 | 4156 |  |
|  | **Variceal bleeding** | | |
| No. of patients | 21580 | 7240 | 28820 |
| No. of patients died in hospital | 2372 | 844 |  |
|  | **Non-variceal bleeding** | | |
| No. of patients | 301357 | 89762 | 391119 |
| No. of patients died in hospital | 9131 | 3312 |  |
| **Myers, 2009** | **Variceal bleeding** | | |
| No. of patients | 27497 | 9237 | 36734 |
| No. of patients died in hospital | 2970 | 1044 |  |
| Time to endoscopy Mean/SD (hours) | 26.9/0.7 | 27.4/0.7 |  |
| **Shaheen, 2009** | **Non-variceal bleeding** | | |
| No. of patients | 180142 | 57270 | 237412 |
| No. of patients died in hospital | 5440 | 1953 |  |
| **Schmulewitz, 2005** | **UGIB** | | |
| No. of patients | 440 | 144 | 584 |
| No. of patients died in hospital | 19 | 9 |  |
