# Supplementary material for: Weekend effect in upper gastrointestinal bleeding: a systematic review and meta-analysis
Source: PeerJ. 2018 Jan 12;6:e4248. doi: 10.7717/peerj.4248 (PMC5768163; doi:10.7717/peerj.4248)
Supplement: Article S1 — 1. The rationale for conducting the meta-analysis; 2. The contribution that the meta-analysis makes to knowledge in light of previously published related reports, including other meta-analyses and systematic reviews. [file peerj-06-4248-s005.pdf]

1. The rationale for conducting the meta-analysis

Upper gastrointestinal bleeding (UGIB) is a significant medical condition worldwide and is the most common reason for which gastroenterologists are consulted (Khamaysi & Gralnek 2013).

Weekend effect, defined as increased mortality in patients who are admitted on the weekend (Bell & Redelmeier 2001; Cram et al. 2004), is suggested to play an important role in patients with UGIB due to several reasons, including reduced staff availability, lack of gastrointestinal subspecialists to conduct timely diagnostic or therapeutic procedures, inadequate intensive care facilities (Zhou et al. 2016) and the fact that sicker patients tend to be admitted on the weekend (Shaheen et al. 2009).

The results reported in the literature are conflicting regarding the association between weekend admission and mortality in patients with UGIB (Abougergi et al. 2014; Ahmed et al. 2015; Dorn et al. 2010; Tufegdzic et al. 2014). Previous meta-analysis explored the weekend effect of UGIB but did not further differentiate different etiologies of UGIB nor the timing of endoscopic intervention (Zhou et al. 2016). Thus we conduct our meta-analysis to investigate the weekend effect of UGIB and analyze important clinical factors that can help devise better program to diminish the weekend effect.

2. The contribution that the meta-analysis makes to knowledge in light of previously published related reports, including other meta-analyses and systematic reviews.

Our study is the first systematic review and comprehensive meta-analysis to address the issue of whether a global weekend effect is associated with mortality in patients with UGIB. We found a significant weekend effect on mortality, a finding that is consistent with several nationwide studies in the United States and population-based studies in the United Kingdom (Ahmed et al. 2015; Button et al. 2011; de Groot et al. 2012; Dorn et al. 2010; Weeda et al. 2016). We also found that the weekend effect was associated mainly with non-variceal bleeding. In addition, pooled data of time to endoscopy during weekday admission showed significantly shorted times than for

weekend admission, indicating that timing of endoscopy may be a critical factor. The timing to endoscopic examination and appropriate intervention may be important factors that influence mortality in UGIB patients. Our analysis provides more comprehensive and detailed knowledge in understanding this significant association and improving patient management.
